# Supplementary material for: Tracheostomy in COVID-19 acute respiratory distress syndrome patients and follow-up: A parisian bicentric retrospective cohort
Source: PLoS One. 2021 Dec 22;16(12):e0261024. doi: 10.1371/journal.pone.0261024 (PMC8694414; doi:10.1371/journal.pone.0261024)
Supplement: S1 Table — Abbreviations: BMI = body mass index; COVID-19 = coronavirus infectious disease-19; ECMO = extracorporeal membrane oxygenation; ICU = intensive care unit; IQR = interquartile range; MV = mechanical ventilation; PCR = polymerase chain reaction; PEEP = positive end-expiratory pressure SAPS 2 = severity acute physiologic score 2. (DOCX) [file pone.0261024.s001.docx]

**Supplemental Material**

**Supplemental Table 1**: Patients characteristics during intensive care unit hospitalization outcomes after intensive care unit hospitalization with surgical and percutaneous tracheostomy.

.

| **Variables** | **All patients**  **(N=48)** | **Surgical tracheostomy**  **(n=24)** | **Percutaneous tracheostomy**  **(N=24)** | **p-value** |
| --- | --- | --- | --- | --- |
| **Demographics** | | | | |
| Age, mean [IQR] - yr | 56 [47-65] | 53 [48-66] | 60 [46-64] | 0.842 |
| Male - no. (%) | 36 (75) | 16 (67) | 20 (83) | 0.317 |
| BMI, median [IQR] - kg/m^2^ | 29 [27-33] | 31 [27-33] | 28 [27-30] | 0.130 |
| BMI > 30 kg/m^2^ - no. (%) | 21 (44) | 15 (63) | 6 (25) | 0.020 |
| Chronic disease - no. (%)   - Chronic heart disease - Chronic kidney disease - Asthma - Obstructive sleep apnea - Immunosuppression | 6 (13)  4 (8)  6 (13)  4 (8)  6 (13) | 2 (8.3)  4 (17)  3 (13)  2 (8.3)  3 (13) | 4 (17)  0 (0))  3 (13)  2 (8.3)  3 (13) | 0.663  0.117  1  1  1 |
| Pregnancy - no. (%) | 1 (2) | 0 (0) | 1 (4.2) | 1 |
| Cardiovascular risk factors - no. (%)   - Hypertension - Diabetes mellitus - Current smoker | 23 (48)  14 (29)  11 (23) | 11 (46)  7 (29)  8 (33) | 12 (50)  7 (29)  3 (13) | 1  1  0.170 |
| **COVID-19** | | | | |
| Symptoms to hospital admission, median [IQR] - days | 7 [5-8] | 6 [3-8] | 7 [5-9] | 0.176 |
| Hospital admission to MV, median [IQR] - days | 2 [1-3] | 2 [1-4] | 1 [0-3] | 0.848 |
| **Characteristics in ICU** | | | | |
| SAPS 2 - mean [range] | 43 [20-74] | 44 [26-72] | 42 [20-74] | 0.800 |
| Mechanical ventilation   - Neuromuscular blockades  - no. (%) - Prone positioning  - no. (%) - ECMO  - no. (%) | 48 (100)  32 (67)  6 (13) | 24 (100)  17 (71)  3 (13) | 24 (100)  15 (63)  3 (13) | 1  0.760  1 |
| Organ dysfunction during ICU stay   - Vasopressors  - no. (%) - Renal replacement therapy  - no. (%) | 48 (100)  14 (29) | 24 (100)  8 (33) | 24 (100)  6 (25) | 1  0.751 |
| Patients with specific treatments for COVID-19   - Hydroxychloroquine - no. (%) - Steroids - no. (%) - Lopinavir/ritonavir - no. (%) - Anakinra - no. (%) - Tocilizumab - no. (%) - Remdesivir - no. (%) | 24 (50)  1 (2)  14 (29)  15 (31)  2 (4)  2 (4)  2 (4) | 17 (71)  0 (0)  12 (50)  11 (46)  1 (4.3)  3 (13)  1 (4.3) | 7 (29)  1 (4.3)  2 (8.3)  4 (17)  1 (4.3)  2 (8.3)  0 (0) | 0.009 |
| Tracheostomy | | | | |
| Early tracheostomy (%) | 10 (21) | 4 (17) | 6 (25) | 0.722 |
| Delay from MV, median [IQR] - days | 17 [12-22] | 17 [12-20] | 16 [11-30] | 0.189 |
| Discharge and vital status | | | | |
| Discharged alive from ICU - no. (%) | 43 (90) | 21 (88) | 22 (92) | 1 |
| Discharged alive from hospital - no (%) | 41 (85) | 20 (83) | 21 (88) | 1 |
| Returned home - no (%) | 41 (85) | 20 (83) | 21 (88) | 1 |
| ICU length of stay, days |  |  |  |  |
| *All patients* | 32 [18-47] | 35 [20-46] | 27 [16-47] | 0.449 |
| *Surviving patients* | *N=25*  31 [18-46] | *N=20*  35 [20-45] | *N=21*  26 [16-46] | 0.195 |
| Hospital length of stay, days |  |  |  |  |
| *All patients* | 48 [24-61] | 52 [28-69] | 35 [22-56] | 0.303 |
| *Surviving patients* | *N=41*  45 [26-60] | *N=20*  55 [34-73] | *N=21*  35 [23-55] | 0.070 |
| Time from hospital admission to home return, days | *N=41*  66 [39-114] | *N=20*  92 [61-118] | *N=21*  44 [34-81] | **0.012** |
| Time spent in rehabilitation center, days | *N=41*  35 [14-51] | *N=20*  45 [22-71] | *N=21*  24 [11-38] | **0.045** |
| Ventilation and tracheostomy | | | | |
| Duration of mechanical ventilation, days |  |  |  |  |
| *All patients* | *N=48*  32 [22-41] | *N=24*  35 [30-43] | *N=24*  27 [22-39] | 0.272 |
| *Surviving patients* | *N=41*  34 [25-41] | *N=20*  35 [30-43]] | *N=21*  27 [22-39] | 0.272 |
| Spontaneous breathing on tracheostomy, days |  |  |  |  |
| *All patients* | *N=48*  12 [7-19] | *N=24*  35 [30-43] | *N=24*  27 [22-39] | 0.272 |
| *Surviving patients* | *N=41*  12 [7-19] | *N=20*  17 [12-23]] | *N=21*  9 [5-14] | **0.009** |
| Time on cannula, days |  |  |  |  |
| *All patients* | *N=48*  32 [22-41] | *N=24*  35 [30-43] | *N=24*  27 [22-39] | 0.272 |
| *Surviving patients* | *N=41*  21 [15-34] | *N=20*  25 [19-36] | *N=21*  16 [14-22] | 0.160 |
| Post-tracheostomy complications | | | | |
| Patients with distant ENT exam | N=30 | N=17 | N=13 |  |
| At least one complication | 17 (57) | 11 (65) | 6 (46) | 0.380 |
| *Unilateral laryngeal palsy* | 5 (17) | 4 (24) | 1 (7.7) | - |
| *Dysphonia* | 9 (30) | 5 (29) | 5 (31) | - |
| *Dysphagia* | 6 (20) | 5 (29) | 1 (7.7) | - |
| *Laryngeal sensitivity dysfunction* | 3 (10) | 2 (12) | 1 (7.7) | - |
| *Laryngeal edema* | 4 (13) | 2 (12) | 2 (15) | - |
| *Tracheal stenosis* | 2 (6,7) | 1 (5.9) | 1 (7.7) | - |

Data are expressed in number (%) or median [interquartile range] as appropriate.

*Abbreviations*: BMI= body mass index; COVID-19= coronavirus infectious disease-19; ECMO= extracorporeal membrane oxygenation; ICU= intensive care unit; IQR= interquartile range; MV= mechanical ventilation; PCR= polymerase chain reaction; PEEP= positive end-expiratory pressure SAPS 2= severity acute physiologic score 2.
